# Semisolid Dosage

**DOI:** 10.3390/pharmaceutics12040315

**Published:** 2020-04-01

**Authors:** Dominique Jasmin Lunter, Rolf Daniels

**Affiliations:** Department of Pharmaceutical Technology, Eberhard Karls University, Auf der Morgenstelle 8, 72076 Tuebingen, Germany

Already in ancient times, semisolid preparations for cutaneous application, popularly known as ointments, played an important role in human society. An advanced scientific investigation of “ointments” as dosage forms was set off in the late fifties of the previous century. It was only from then on that the intensive physico-chemical characterization of ointments as well as the inclusion of dermatological aspects led to a comprehensive understanding of the various interactions between the vehicle, the active ingredient, and the skin.

In the meantime, many researchers have been involved in optimizing semisolid formulations with respect to continuously changing therapeutic and patient needs. Aspects that have been dealt with are the optimization of dermato-biopharmaceutical properties and many different issues related to patient’s compliance, such as skin tolerance, applicability, and cosmetic appeal. Moreover, processing technology has been improved and analytical techniques developed and refined in order to enable improved characterization of the formulation itself as well as its interaction with the skin.

This Special Issue serves to highlight and capture the contemporary progress and current research on semisolid formulations such as dermal drug delivery systems. We gathered articles on different aspects of semisolid formulations highlighting the research currently undertaken to improve and better understand these complex drug delivery systems, in particular with respect to formulation, processing, and characterization issues.

This Special Issue comprises 12 articles featuring the various aspects of semisolids, which mainly comprise but are not limited to cutaneous application.

Three papers in this Special Issue deal with formulations intended to treat wounds. In particular, the paper by Ghaffar et al. describes a concept to enhance the gelling ability of an oleogel consisting of sunflower oil and a birch bark extract where the triterpene extract functions as the active substance as well as the gelling agent [1]. In order to save the extract, the authors studied several additives, which can act as linkers between the extract particles and thereby enhance the formation of a particulate network. The most pronounced effect was observed in diols with terminal hydroxyl groups, e.g., 1,6-Hexanediol. In contrast, 1,2-diols impaired gel formation by blocking superficial OH groups on the extract particles.

The contribution of Ternullo et al. describes ‘’Curcumin-In-Deformable Liposomes-In-Chitosan-Hydrogel as a Novel Wound Dressing’’ that utilizes the wound-healing potential of both curcumin and chitosan [2]. Most promising results were achieved with positively charged deformable liposomes. They proved to stabilize the formulation’s bioadhesiveness and allowed sustained permeation of curcumin through ex-vivo fullthickness-human skin. The developed advanced dermal delivery system therefore seems to be a promising candidate as a wound dressing.

As third article on wound healing, Lee et al. present results of ‘’In-Situ Hydrogel-Forming/Nitric Oxide-Releasing Wound Dressing for Enhanced Antibacterial Activity and Healing in Mice with Infected Wounds’’ [3]. The formulation is a dry powder consisting of alginate, pectin, PEG, and S-nitrosoglutathione, which shows good storage stability. When applied to wounds, it absorbs wound fluid and transforms it into an adhesive hydrogel that enables a controlled NO release property for the effective treatment of infected wounds.

The paper of Liu et al. is entitled “Systematic Investigation of the Effect of Non-Ionic Emulsifiers on Skin by Confocal Raman Spectroscopy—A Comprehensive Lipid Analysis” [4]. The article deals with the effect of topically applied emulsifiers on the qualitative and quantitative composition of the stratum corneum lipids. Using confocal Raman spectroscopy (CRS), the authors could demonstrate that polyethylene glycol (PEG) sorbitan esters revealed no alteration of intercellular lipid properties, while PEG-20-ethers appeared to have the most significant effects on reducing lipid content and interrupting lipid organization. Thus, CRS was shown to be a valuable tool to characterize the molecular effects of nonionic emulsifiers on skin lipids and further deepen the understanding of enhancing substance penetration with reduced skin barrier properties and increased lipid fluidity.

Formulation development for treatment of various skin diseases is the topic of the following three papers.

Schmidberger et al. present a study dealing with the ”Optimization of Rheological Behaviour and Skin Penetration of Thermogelling Emulsions with Enhanced Substantivity for Potential Application in Treatment of Chronic Skin Diseases” [5]. The aim of this study was to find an innovative formulation with increased substantivity allowing for a controlled cutaneous drug release, reduced application frequency, and diminished contamination of patients’ environment with the active ingredients. This was achieved by adding high amounts of methyl cellulose to a cream, which changes the formulation into a predominantly elastic body at skin surface temperature.

Berenguer and coworkers present results on a ‘’Topical Amphotericin B (AmB) Semisolid Dosage Form for Cutaneous Leishmaniasis: Physicochemical Characterization, Ex-Vivo Skin Permeation and Biological Activity’’ [6]. The study describes an AmB gel that proved to be stable for 60 days at 4 °C and showed characteristics that made it favorable for cutaneous application. As desired, ex-vivo permeation experiments revealed that neither application to damaged nor to nondamaged skin produced detectable concentration of AmB in the receptor fluid. Furthermore, no cytotoxic effects were observed in the macrophage or in the keratinocyte cell lines. This makes the AmB gel a promising candidate for further evaluation of its activity and efficacy in the treatment of cutaneous leishmaniasis.

Finally, the article of Rancan and coworkers deals with ‘’Dermal Delivery of the High-Molecular-Weight Drug Tacrolimus by Means of Polyglycerol-Based Nanogels’’ [7]. The authors show that tacrolimus formulated as ointment or nanogel suspension penetrates skin with different efficiency in dependence on SC thickness and integrity. Irritation effects of tacrolimus ointment and nanogel formulations, reflected by the released inflammatory cytokines IL-6 and IL-8, were more pronounced in barrier-disrupted and immuno-activated skin. The results support the key role of the SC as barrier for drug and nanocarrier penetration and underline the critical balance of penetration enhancement and potential increase of side effects. All in all, the results suggest that nanogel suspensions are valuable dermal delivery systems for high molecular weight, poorly water-soluble drugs like tacrolimus.

Four of the remaining articles deal with the investigation of skin penetration and permeation.

Zsikó et al. provide a “Comparison of Skin Penetration Testing Methods based on a Nanostructured Lipid Carrier (NLC) Gel for the Dermal Application of Lidocaine” [8]. As expected, drug release profiles of the Lidocaine-NLC gel obtained with the different techniques were not fully equivalent. The various tested synthetic membranes were shown to be useful tools to examine the permeation/release of an active from a dermal formulation. The presented results can be used to guide formulators in selecting appropriate vehicles. However, no general recommendation can be made and it is still a challenging task for researchers to select the most suitable membrane to be used with Franz cells for topical product testing.

Rath et al. present in their article ‘’A Validated In-Vitro Release Test (IVRT) Method to Assess Topical Creams Containing Metronidazole Using a Novel Approach’’ [9]. The reported IVRT method was carefully developed and comprehensively validated to assess the release of metronidazole from cream products, taking into account the various parameters that may affect the API release rate. The presented data indicate that the developed IVRT method can be applied to accurately and precisely assess “sameness” and differences between various metronidazole cream products as a valuable procedure in formulation development.

Zhang et al. summarize in their article results on the ‘’Influence of Binary and Ternary Solvent Systems on the Topical Delivery of Niacinamide’’ [10]. Binary systems consisting of propylene glycol (PG) and some fatty acids showed enhanced skin penetration. However, the correlation for the permeation data of binary and ternary systems in Skin (Parallel Artificial Membrane Permeability Assay) PAMPA and in porcine skin was limited. It could be clearly improved by excluding the PG-Oleic Acid and PG-Linoleic Acid systems, indicating that there is still a lack of knowledge concerning specific interactions between the Skin PAMPA model and penetration-enhancing excipients.

“Preparation, Characterization and Dermal Delivery of Methadone” is the title of the article by Kung et al. [11]. They tested a range of solvents using ex-vivo permeation and mass balance method in porcine skin. Their data identified octyl salicylate, d-limonene, Transcutol^®^, and ethyl oleate as the most promising penetration enhancer for methadone base. Furthermore, maximum skin flux was estimated. Although the study confirms skin permeation of methadone base, the outcome was suboptimal as the majority of the active remained on the skin surface after 24 h under finite dose conditions.

Last but not least, the article that does not deal with dermal application describes a ‘’Mucoadhesive Budesonide Formulation for the Treatment of Eosinophilic Esophagitis (EE)’’ [12]. The authors present results from a study that revealed a standardized budesonide oral formulation intended to improve the resistance time of the drug on the esophageal mucosa for EE treatment. The development focused on the formulation’s physicochemical stability and the main critical quality attributes of the formulation, e.g., rheological properties, syringeability, mucoadhesiveness, and in-vitro penetration of budesonide in porcine esophageal tissue. The optimized formula demonstrated that the used gums enable a prolonged residence time in the esophagus.

The purpose of this Special Issue was to provide an overview of recent advances in the field of semisolid formulations. Following the results of the interesting articles collected for this Special Issue, we can conclude that although semisolids already played an important role in human society in ancient times there is still innovative research in this field adding new pieces to the jigsaw puzzle.
